# Harnessing Nanomaterials for Water Decontamination: Insights into Environmental Impact, Sustainable Applications, and the Emerging Role of Polymeric Nanostructures

**DOI:** 10.3390/polym18030393

**Published:** 2026-02-02

**Authors:** Tony Hadibarata, Risky Ayu Kristanti, Adelina-Gabriela Niculescu, Dana-Ionela Tudorache (Trifa), Alexandra Cătălina Bîrcă, Alexandru Mihai Grumezescu

**Affiliations:** 1 Environmental Engineering Program, Faculty of Engineering and Science, Curtin University Malaysia, CDT 250, Miri 98009, Malaysia; hadibarata@curtin.edu.my; 2 Department of Science and Engineering of Oxide Materials and Nanomaterials, National University of Science and Technology POLITEHNICA Bucharest, 011061 Bucharest, Romania; adelina.niculescu@upb.ro (A.-G.N.); dana.tudorache@upb.ro (D.-I.T.); alexandra.birca@upb.ro (A.C.B.); 3 Research Center for Oceanology, National Research and Innovation Agency, Jalan Raya Puspiptek No. 60, Tangerang Selatan 15314, Indonesia; risky.ayu.kristanti@brin.go.id; 4 Research Institute of the University of Bucharest—ICUB, University of Bucharest, 90–92 Panduri, 050663 Bucharest, Romania

**Keywords:** nanomaterials, water decontamination, environmental impact, toxicity assessment, sustainable strategies

## Abstract

Nanomaterials provide novel solutions for water treatment because of their unique properties and functions, such as a large surface area, increased reactivity, and interaction with contaminants at the nanoscale. These useful features make nanomaterials highly effective in addressing water-related issues, especially in the remediation of aquatic environments from heavy metals, organic pollutants, and microplastics. However, there are increasing concerns about their persistence in the environment and the possible risks to ecosystems and human health, due to their tendency to bioaccumulate and enter food chains. While some nanomaterials have proven toxic even at low concentrations, most effects that these materials may have on aquatic organisms, plants, and animals remain largely unexplored. Most sources report that polymeric nanomaterials are also the least toxic and most environmentally compatible, particularly when biodegradability forms one of the design parameters. Polymeric nanoparticles can be considered a safer alternative to metal- and carbon-based nanomaterials. However, they can not be used without any risk at all. The long-term environmental accumulation of nanoplastics and their potential chronic ecological impacts have received greater attention recently. This paper reviews major research on the toxicity and environmental behavior of nanomaterials, with a special focus on their long-term ecological effects, for which substantial knowledge exists, yet highlights gaps in existing knowledge and future directions for responsible application in water treatment contexts.

## 1. Introduction

Water pollution is a major challenge to the environment and human health. Heavy metals (such as lead, mercury, and arsenic), organic pollutants (including pesticides, pharmaceuticals, and industrial chemicals), and particulate/physical pollutants (such as microplastics) contaminate water bodies [1,2,3,4]. Heavy metals are highly toxic and persistent in the environment, and therefore bioaccumulate in many organisms. The same principle also applies to organic pollutants, which disrupt aquatic ecosystems and human health [2]. Another emerging, widespread pollutant is microplastics, which have negative effects on marine life and can potentially enter human food chains [5]. While conventional water treatment techniques have proven effective, they are not without substantial limitations. Heavy metals, organic pollutants, and microplastics can be difficult to remove through filtration and chemical treatments [6]. For example, flocs produced with conventional coagulants might not remove dissolved contaminants or trace levels of organic pollutants. This method also does not guarantee a complete degradation of microplastics [7]. A previous study showed that traditional technologies produce chemical by-products or residues that require disposal, creating secondary environmental problems [8]. Consequently, newer, more advanced technologies are required to meet the evolving challenges.

Nanomaterials are positioned as advanced water treatment solutions, demonstrating undoubted superiority over conventional systems. Special characteristics of nanomaterials, such as high surface area, increased reactivity, and the ability to interact with contaminants at the nanoscale, provide high effectiveness in addressing problems related to aquatic pollution from heavy metals, organic pollutants, or microplastics [9,10]. The greatest advantage is their exceptional efficiency in dealing with contaminants. Due to their high surface area, multiple active sites facilitate adsorption or interaction with contaminants, resulting in faster and complete water purification. Nanomaterials can also be designed to remove specific pollutants [11]. This effect is often impossible to achieve using routine methods. For instance, certain nanomaterials can be tailored to selectively capture harmful heavy metals or degrade persistent organic compounds, thereby enhancing the accuracy of the purification process [10,11].

Additionally, the multifunctional nature of nanomaterials presents a significant benefit. In contrast to conventional water treatment methods, which typically involve multiple stages such as filtration, chemical disinfection, and contaminant degradation, nanomaterials can perform all these processes in a single step. This reduces operational complexity, energy consumption, and costs. Furthermore, because most nanomaterials produce few hazardous by-products, they are significantly more environmentally friendly than conventional chemicals [11,12]. Furthermore, certain nanomaterials, including metal oxides, can be recovered and reused multiple times, thereby enhancing their sustainability. Common instances of nanomaterials employed in the treatment of drinking water include carbon nanotubes (CNTs), nanoscale zero-valent iron (nZVI), and titanium dioxide nanoparticles (TiO_2_). CNTs effectively absorb heavy metals, organic pollutants, and even pathogens. nZVI has wide applications for the effective removal of heavy metals such as arsenic and lead, and for simultaneously decomposing organic contaminants. TiO_2_ nanoparticles, on the other hand, are utilized in the photocatalytic degradation of organic pollutants and the conversion of harmful compounds into less harmful substances under UV irradiation. These versatile nanomaterials hold great promise for providing clean, cost-effective water treatment solutions [13,14].

Understanding the environmental toxicity implications of nanomaterials is crucial, given their increasing use in water treatment and other fields. The improved application of nanomaterials raises significant concerns about their persistence and potential risks to ecosystems, including human health. Nanomaterials readily infiltrate the composition of aquatic systems, soil, and even the atmosphere. Over time, bioaccumulation within living organisms or food chains can pose a concern. The effects of nanomaterial exposure in ecosystems remain to be established, as bioaccumulation and toxicity to aquatic life, plants, and animals are significant concerns. Research indicates that nanomaterials, including metal nanoparticles, can induce oxidative stress, cause cellular damage, and exhibit toxicity to organisms, even at low concentrations [15,16].

This persistence of nanoparticles and their potential risks highlight the necessity for thorough investigations into their environmental impacts. Key factors, such as degradability, reactivity with environmental components, and effects on different species, determine the appropriate conditions for their safe use. According to this priority, there is also a critical need to relate appreciable activity in such applications as water treatment to the environmental safety of the nanomaterials. While many nanomaterials demonstrate outstanding short-term performance, their long-term ecological consequences must be carefully weighed against these immediate benefits [14,16].

Within this context, growing attention has been directed toward nanomaterials that can be intentionally designed to minimize persistence and toxicity. In particular, polymeric nanomaterials occupy a distinctive and increasingly important position, as their chemical versatility enables the tuning of surface functionality, degradation pathways, and interaction with biological systems [17]. Compared with many inorganic or carbon-based counterparts, polymer-based nanostructures offer greater opportunities for incorporating biodegradability and reduced ecotoxicity into material design, aligning water treatment performance with environmental compatibility [18].

In this paper, nanomaterials are defined as materials with at least one dimension in the 1–100 nm range. Nanoparticles are considered a specific subclass of nanomaterials in which all three external dimensions fall within this nanoscale range. The environmental and ecotoxicological mechanisms discussed herein—such as dispersion, aggregation, and sedimentation in aquatic systems—are relevant to dispersible and particulate nanomaterials, including both nanoparticles and high-aspect-ratio nanostructures. In contrast, bulk materials that merely exhibit nanoscale surface features but lack particulate or dispersible behavior are outside the scope of this analysis.

The novelty of this article lies in the combined evaluation of the transport and fate of nanomaterials in water environments, along with their ecological toxicity in these environments, an aspect typically addressed separately in the existing literature. Based on the integrated perspective, the objective of this review is to critically evaluate the current research on the environmental behavior and ecological effects of nanomaterials, while recognizing key knowledge gaps and future research aimed at ensuring their long-term safety and effectiveness.

## 2. Categories of Nanomaterials Utilized in Water Decontamination

**Figure 1:**
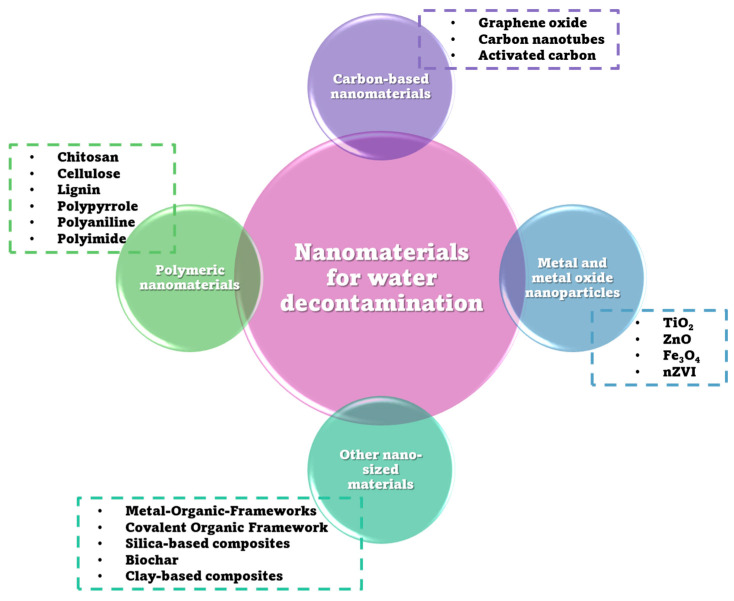
Schematic overview of nanomaterial categories for water decontamination. nZVI—nanoscale zero-valent iron.

Nanomaterials utilized for the decontamination of water can be classified into several categories: carbon-based nanomaterials, metal and metal oxide nanoparticles, polymeric nanomaterials, and various other nano-sized materials (Figure 1). Each of these categories provides unique mechanisms and benefits for pollutant removal, which will be elaborated upon in the subsequent subsections.

### 2.1. Carbon-Based Nanomaterials

Carbon-based nanomaterials (CBNs) have emerged as the most effective substances for water treatment, attributed to their distinctive adsorption properties and substantial BET surface area [19,20]. Among these materials are graphene oxide (GO) [21], CNTs [22], and activated carbon (AC) [23] (Table 1). These substances utilize interactions such as van der Waals forces, π-π stacking, hydrogen bonding, and electrostatic interactions to adsorb a diverse range of pollutants. Heavy metals, organic pollutants, and other contemporary contaminants exhibit significant increases in adsorption onto such nanomaterials, thereby highlighting their integral roles in advanced water treatment. The performance characteristics of each material, nonetheless, are influenced by its surface chemistry and structural attributes. This, in turn, implies the importance of understanding the adsorption mechanisms and efficiencies in real-world water purification systems [20].

#### 2.1.1. GO

GO is a chemically modified form of graphene created by introducing various oxygen-functional groups (such as hydroxyl, epoxy, and carboxyl), which make it more hydrophilic and more effective for water purification [21,24]. In this context, these groups help in the adsorption of a wide spectrum of contaminants, including heavy metals such as copper, iron, and arsenic, as well as organic dyes and pharmaceuticals. A particular advantage of GO is the ultra-high surface area and the flexibility for functionalization to specific pollutants. In addition, GO is highly water-soluble; hence, it interacts well with impurities in the water phase [25]. The issue with GO is its aggregative behavior under certain conditions, which decreases the available surface area and adsorption efficiency. Also, producing GO can be expensive and may require further research to make it economically viable at larger scales for different commercial applications [25,26].

#### 2.1.2. CNTs

CNTs are graphene sheets rolled into cylindrical structures, distinguished by their exceptional electronic and mechanical properties. They are divided into two main types, namely single-walled CNTs (SWCNTs) and multi-walled CNTs (MWCNTs), both of which have been the subject of extensive research regarding their potential applications in water treatment [22,27,28]. The adsorption capacity of CNTs is particularly used for the elimination of organic pollutants, including aromatic hydrocarbons, synthetic dyes, and insecticides, as well as heavy metals such as copper, iron, and mercury, owing to their efficient adsorption mechanisms [17,29]. The removal of non-polar organic compounds by CNTs is notably effective, as functionalization of the nanotubes imparts a degree of hydrophobicity. Additionally, oxidative treatments facilitate the removal of high-energy sites on the carbon surface, thereby introducing carboxyl or hydroxyl groups [27,28]. An advantageous aspect of this technology is its retention of mechanical strength and stability over time, allowing for easy reuse and reapplication across multiple treatment cycles. Furthermore, CNTs exhibit high adsorption capacities for a wide range of pollutants due to their extensive surface areas and robust adsorbate–adsorbent interactions. However, the hydrophobic nature of CNTs can sometimes pose challenges for direct application in water treatment, as it can lead to inadequate dispersion in aqueous environments. Given that these materials are very expensive, the environmental toxicity they pose, as well as their reusability issues, remain worrisome [30,31].

#### 2.1.3. AC

AC is a widely used water treatment material and is typically found in filtration systems because it is very porous and has a high surface area. Usually, AC is derived from coal, wood, coconut shells, etc., but its adsorptive capacity is enhanced through processing. AC is highly efficient at adsorbing organic pollutants (e.g., volatile organic compounds, VOCs) and some heavy metals [23]. Normally, AC mainly involves van der Waals forces. Adsorption is a physical process, but some polar pollutants can enable chemisorption as well. Throughout its porosity, the majority of contaminants are held within the carbonaceous structure. Major advantages include availability, low-cost production, and proven efficiency as a purifying agent for water. It is prepared in abundance and can be recycled. However, AC still has its limitations to this day; it lags behind in adsorption capacity compared to more advanced nanomaterials such as GO and CNTs. The effectiveness of AC in adsorbing new contaminants, such as pharmaceuticals and microplastics, may not be as high as that of GO. The regeneration process may lead to gradual material degradation, later manifesting as reduced performance [32,33].

**Table 1:** Overview of carbon-based nanomaterials for water treatment.

Type of Nanomaterial	Adsorption Mechanism	Advantages	Limitations	Refs.
GO	Pollutants are primarily captured through electrostatic interactions involving functional groups and ionic species, hydrogen bonding with polar contaminants, and van der Waals forces for non-polar compounds. The presence of oxygen-containing functional groups, such as carboxyl, hydroxyl, and epoxy, increases the affinity for a range of pollutants, including heavy metals, dyes, and pharmaceuticals.	An exceptionally high specific surface area provides a wealth of active sites for adsorption. Exhibit high dispersibility in aqueous environments due to surface oxygen functionalities. Demonstrate effectiveness in the removal of both organic and inorganic pollutants. Can be modified or integrated with other nanomaterials to customize selectivity and enhance performance.	Susceptible to aggregation in solution with high ionic strength or as time progresses, leading to diminished surface availability and reduced adsorption efficiency. Large-scale implementation is constrained by high production costs and complex synthesis processes. The recovery of treated water poses challenges without the necessity for supplementary separation processes.	[9,24,25,26]
CNTs	Employ van der Waals forces and hydrophobic interactions for the adsorption of organic pollutants. Electrostatic attractions are involved when CNTs are functionalized or oxidized. Their hollow, tubular structure and large aspect ratio provide unique adsorption pathways and trapping of pollutants inside or on the tube walls.	Their remarkable mechanical strength and structural integrity enable reuse across multiple cycles. A significant surface area, combined with π–π interactions, results in robust affinity for a range of organic contaminants. Capable of undergoing chemical modifications to improve dispersibility and selectivity. Demonstrate potential for advanced oxidation processes and catalytic degradation.	Native hydrophobicity limits dispersion in aqueous environments unless functionalized, reducing adsorption potential. Production is still relatively expensive, especially for SWCNTs. The potential for environmental and human toxicity arises from biopersistence and the ability to penetrate cellular membranes. The uncertainty surrounding the risk of bioaccumulation and its long-term ecological consequences persists.	[22,27,28,30,31]
AC	Utilizes physical adsorption, specifically van der Waals forces, to capture pollutants within pores and employs chemisorption through surface functional groups to create more robust and frequently irreversible interactions. Demonstrate efficacy against a diverse array of organic molecules, particularly within neutral to slightly acidic pH levels.	Economical and readily accessible from natural sources, including coconut shells, coal, or wood. Established application in commercial water filtration systems. Significant pollutant absorption is facilitated by high porosity and surface area. Capable of being thermally or chemically regenerated for subsequent use.	Exhibits a relatively lower adsorption capacity for specific emerging or highly polar contaminants. It becomes saturated in a relatively short period, necessitating frequent regeneration or replacement. Efficiency decreases after multiple regeneration cycles due to pore collapse or surface fouling. Less effective for some metal ions without chemical modification.	[23,32,33]

Abbreviations: GO—graphene oxide, CNTs—carbon nanotubes, SWCNTs—single-walled CNTs, AC—active carbon.

### 2.2. Metal and Metal Oxide Nanoparticles

Metal/metal oxide nanoparticles, including TiO_2_ [34], zinc oxide (ZnO) [35], and nZVI [36], are gaining popularity because they are highly reactive, have high surface areas, and can contribute to advanced oxidation and/or redox processes for water treatment applications (Table 2). These materials can degrade most organic pollutants and remove heavy metals from the aquatic environment. The working principles include photocatalytic degradation, redox reactions, and adsorption, which are highly effective in combating complex pollution, water disinfection, and pollutant removal [37].

#### 2.2.1. TiO_2_ Nanoparticles

Among metal nanoparticles, TiO_2_ is widely used in photocatalysis because it is chemically and physically stable and therefore biologically friendly. TiO_2_ has been used to eliminate practically all forms of organic pollutants, such as synthetic dyes, aromatic hydrocarbons, pesticides, and VOCs [38,39]. When subjected to ultraviolet (UV) irradiation, TiO_2_ generates highly reactive oxygen species (ROS) that facilitate the breakdown of pollutants into water and carbon dioxide. Among these species are hydroxyl radicals (•OH) and superoxide anions (O_2_•−). Most importantly, the catalyst is durable, steady, and non-hazardous; moreover, it can be recovered and reused in numerous cycles and performs well in both organic and inorganic pollutions [34,38]. On the other hand, TiO_2_ requires UV light to exhibit photocatalytic activity, which limits its application under natural sunlight, as only about 5% of sunlight is UV. It is for this very reason that natural photocatalysts normally work well with TiO_2_, requiring the present system to be carried out under UV light. Better visible-light responsiveness is actively pursued by scientists through alternative forms of TiO_2_, such as doping with other elements, such as nitrogen or silver [39,40].

#### 2.2.2. ZnO Nanoparticles

ZnO is one more metal oxide nanoparticle that can possess photocatalytic as well as antibacterial activity. Similarly to TiO_2_, ZnO can generate ROS under UV light that aid in the degradation of many organic pollutants, such as dyes, phenolic compounds, and pharmaceutical residues [35,41,42]. Another noted function of ZnO is water disinfection via the rupture of bacterial and other microorganism cell walls. One advantage of using ZnO is its high photocatalytic efficiency and its similar applications to TiO_2_, but with a larger bandgap, leading to greater ROS generation. It exhibits high antimicrobial activity and is suitable for water treatment disinfection. In the same way as TiO_2_, ZnO is primarily operative only under UV light and is also sometimes borne into question for its stability during longer exposure because it dissolves in acidic or high ionic strength environments; in contrast, ZnO nanoparticles themselves may be toxic to aquatic life and should be considered in the context of environmental effects and accumulation in the ecosystem [42,43].

#### 2.2.3. Fe_3_O_4_ Nanoparticles

The use of magnetic nanoparticles, particularly magnetite (Fe_3_O_4_), has been widely explored for water decontamination. Magnetite is the most naturally occurring magnetic mineral, characterized by ease of synthesis, colloidal stability, dispersibility, functionalization possibility, and eco-friendliness [17,44]. These nanostructures offer significant benefits by facilitating magnetic separation, recovery, and reuse, thereby reducing the required materials and the duration of water treatment processes [29]. Their high surface area and magnetic properties enable effective adsorption of heavy contaminants and easy separation from aqueous solutions. Fe_3_O_4_ nanoparticles exhibit a strong affinity for heavy metals like lead (Pb^2+^) and chromium (Cr^6+^), mainly through surface complexation and electrostatic interactions [45,46]. Fe_3_O_4_ nanoparticles can be combined with a wide range of materials to form various magnetic nanoadsorbents suitable for the effective removal of heavy metals and organic pollutants from contaminated water samples [17,47,48,49]. Furthermore, they can be used to fabricate recyclable, magnetically separable photocatalysts for antibiotic degradation [50,51]. For example, Ag-Fe_3_O_4_ nanocomposites can generate ROS that degrade organic compounds and exhibit antibacterial properties, thereby enhancing disinfection in wastewater treatment [51,52]. Additionally, Fe_3_O_4_ nanoparticles have been employed to remove microplastics from water bodies, achieving over 80% removal efficiency by magnetizing the microplastics for subsequent magnetic separation [53]. Nonetheless, since most Fe_3_O_4_ nanocomposite adsorbents have been tested only at the laboratory scale, additional studies are needed to assess their large-scale environmental impact and reduce potential secondary pollution [17].

#### 2.2.4. nZVI

A commonly used metal nanoparticle for the remediation of groundwater and wastewater contaminated with heavy metals is nanoparticle nZVI. It primarily acts as a potent reagent for heavy metals and chlorinated organic compounds via redox reactions [36,54,55]. nZVI supports reducing contaminants to less harmful conditions or to immobile forms that can then be removed through water treatment technologies, based on the process principle. Furthermore, nZVI can mineralize complex organics into simpler, less harmful molecules. Its key advantages are high reactivity against a widespread range of contaminants and comparatively low cost. It is also effective for removing heavy metals and persistent organic pollutants. However, the main problem that has always limited nZVI application is its tendency to agglomerate, resulting in a reduction in surface area and reactivity. Other concerns about nZVI include its long-term environmental impacts and its potential to move through the underground environment, posing a threat to underground ecosystems. They still constitute a serious habitat disturbance in the environment [56,57].

**Table 2:** Overview of metal and metal oxide nanoparticles for water treatment.

Type of Nanoparticle	Mechanism of Pollutant Removal	Advantages	Limitations	Refs.
TiO_2_	Photocatalytic degradation via ROS generation under UV light	Exhibits strong photocatalytic activity in breaking down a wide variety of organic pollutants, including dyes, pharmaceuticals, and endocrine disruptors. Exhibiting chemical stability across a broad spectrum of pH levels and temperature conditions, alongside significant resistance to both corrosion and photo-corrosion. Characterized by biocompatibility and non-toxicity, making it appropriate for applications with minimal health risks. Capable of being utilized across several cycles with minimal degradation in performance, thereby lowering operation expenses.	Needs UV light for activation because ot its broad bandgap (~3.2 eV), which constrains efficiency when exposed to solar or indoor lighting. Unless modified through doping, which can increase costs and potentially diminish long-term stability, there is inadequate responsiveness within the visible light spectrum. In aqueous environments, aggregation occurs, leading to a decrease in effective surface area and catalytic efficiency. The recovery of treated water in slurry systems can present significant challenges.	[34,39,40]
ZnO	The generation of ROS facilitates photocatalytic degradation and exhibits antibacterial activity.	Demonstrating comparable, if not superior, photocatalytic performance to TiO_2_, this material benefits from enhanced quantum efficiency and a broader bandgap of ~3.3 eV. It possesses significant antibacterial properties, making it effective for water disinfection and contaminant removal. Economical and readily accessible, it can be produced in a range of nanostructures (e.g., rods, sheets, spheres) to optimize performance. The substantial surface area facilitates rapid electron-transfer reactions, enhancing the degradation of pollutants.	Like TiO_2_, it is restricted to UV light activation and exhibits reduced efficiency under visible light unless modified. Tends to dissolve in water under acidic or high-ionic-strength conditions, potentially releasing Zn^2+^ ions, which may be toxic to aquatic life. Can exhibit photo-corrosion over time, especially in the presence of water and light. Limited stability in certain environmental conditions results in a short functional life without protective coatings.	[35,41,42,43]
Fe_3_O_4_	Affinity for heavy metals through surface complexation and electrostatic interactions In composites, photocatalytic degradation and antibacterial activity via ROS generation	Benefit from easy synthesis routes, cost-effectiveness, and eco-friendliness Exhibit a strong affinity for heavy metals Can be incorporated into composites, bringing synergistic photocatalytic performance and superior pollutant removal efficiency Enable multiple cycles of magnetic separation, recovery, and reutilization, reducing operating costs.	The tendencies for oxidation and agglomeration in their unmodified state necessitate surface functionalization to preserve their long-term efficacy. Certain environmental conditions result in reduced stability, leading to a shorter functional lifespan without surface modification. The long-term ecological impacts of the various composites remain largely unexplored.	[17,44,45,46,51,52,53]
nZVI	Reactions involving the reduction–oxidation process facilitate the reduction in heavy metals and the degradation of organic pollutants	The high surface area and zero-valent state contribute to their significant reactivity, allowing for the swift breakdown of chlorinated organic compounds, nitrates, and heavy metals. These methods are economically viable for extensive environmental remediation efforts, including in situ treatment of groundwater. Adaptable for the remediation of both organic and inorganic pollutants across various water matrices. Facilitate the prolonged sequestration of specific metals by precipitating them as insoluble oxides or hydroxides.	The rapid oxidation that occurs in the presence of water and air can diminish reactivity; therefore, stabilization is essential to preserve effectiveness. Strong tendency to aggregate, lowering dispersibility and reactive surface area in environmental applications. Limited mobility in subsurface environments restricts its use in some field-scale remediation efforts unless surface-functionalized. Potential for secondary contamination or unknown long-term ecological impacts due to iron oxides and by-products.	[36,54,55,56,57]

Abbreviations: nZVI—nanoscale zero-valent iron; ROS—reactive oxygen species; UV—ultraviolet.

### 2.3. Polymeric Nanomaterials

Polymeric nanomaterials are gaining increasing interest among scientists, who see in them, due to their flexibility and adjustability, a promise for various water purification applications (Table 3). At the very basic level, these materials can be endowed with numerous chemical groups to amplify their selectivity and efficiency in capturing targeted contaminants; therefore, they are very easily manipulated. A promising development is the use of functionalized polymeric nanoparticles explicitly designed for targeted contaminant capture [58,59]. The general mechanisms underlying the removal of contaminants by polymeric composites include physical adsorption, ion exchange, and chemical bonding. These interactions with pollutants depend on the nature of functional groups attached to polymers.

#### 2.3.1. Chitosan

Chitosan is a linear, biodegradable, natural polymer widely used in water treatment due to its exceptional chemical versatility, low cytotoxicity, ease of preparation and functionalization, water solubility, polyelectrolyte character, and biocompatibility [60,61]. Due to its rich surface chemistry, dominated by reactive amino and hydroxyl groups, chitosan can be used to attract various classes of contaminants, providing a wide range of applications in wastewater treatment [60,62,63]. Chitosan has also been incorporated into a variety of nanostructures to enhance the adsorption of organic and inorganic contaminants [60,64]. For example, chitosan-magnetic nanocomposites combine chitosan’s ability to bind contaminants with nanoparticles’ properties (large surface area, easy regeneration, and dispersed nature), which have been shown to provide excellent adsorption of rare-earth metals, heavy metals, and dyes [63]. Another application of functionalized chitosan is attributed to its attractive antimicrobial properties, which are valuable for membranes used in industrial desalination from wastewater systems [61]. Furthermore, the incorporation of ZnO inorganic filler into chitosan matrices enhances the composition’s adsorption capacity, resistance to fouling, and overall membrane durability [65].

#### 2.3.2. Cellulose

Cellulose is a natural polymer abundantly available and distinguished by its intrinsic biocompatibility, renewability, and biodegradability. These attributes have garnered attention for their application in the bioremediation of polluted waters, presenting a sustainable alternative to synthetic materials [66]. In its nanoscale form, nanocellulose has found significant utility in water purification, acting as an environmentally friendly adsorbent with a high surface area and chemical versatility [67,68]. Furthermore, patent literature has identified nanocellulose as a “green” carrier for catalysts and other active substances, underscoring its importance in industrial applications and its potential for scalable commercialization [69]. Additionally, cellulose can be functionalized with oleophilic or hydrophobic groups to facilitate oil adsorption or combined with biopolymers (such as chitosan and sodium alginate) and nanomaterials (including noble metals, carbon, CNTs, GO, zeolites, and metal–organic frameworks, MOFs) to enhance mechanical strength and adsorption efficiency. For instance, carboxylated cellulose nanocrystal–alginate beads demonstrate high affinity for toxic heavy metals [70].

#### 2.3.3. Lignin

Lignin is a racemic aromatic heteropolymer produced in plant cell walls by oxidative coupling of three hydroxycinnamyl alcohol monomers (i.e., p-coumaryl, coniferyl, and sinapyl). Previous reports have shown that lignin-based gels and porous lignin beads are feasible and efficient for bioremediation of contaminated water [66]. Lignin has also been investigated as a precursor for carbonaceous materials, including biochar and activated carbon, which provide synergistic physisorption and chemisorption capabilities for the removal of various pollutants. Furthermore, due to its aromatic, polyphenolic structure and high carbon content, lignin can be used to develop photocatalysts with improved properties [71]. Lignin-based nanomaterials, in particular, have emerged as effective biowaste-derived materials with adjustable physicochemical properties suitable for wastewater treatment [72]. The amorphous aromatic framework of lignin facilitates the binding of both organic and inorganic pollutants, while nanocomposites composed of lignin and metal or metal oxide enhance catalytic and adsorption efficacy at reduced cost [72,73].

#### 2.3.4. Other Polymers

Another emerging pathway in environmental remediation is the use of synthetic polymers, which have attracted considerable interest due to their tunable physicochemical properties, molecular structures, and high affinity toward a broad spectrum of contaminants. Among the various materials studied, polypyrrole, a conducting polymer characterized by π-conjugation, has been investigated for its capacity to adsorb both inorganic and organic pollutants, in addition to its remarkable conductivity, environmental resilience, excellent carrier mobility, and ability to absorb full-spectrum light in water treatment applications [74,75]. When combined with graphene-based aerogels, polypyrrole forms π–π stacking interactions that inhibit graphene self-aggregation, leading to the formation of three-dimensional porous hybrid structures. These structures promote pollutant adsorption and improve the separation of photogenerated electron–hole pairs, thus enhancing the efficiency of photocatalytic degradation [75]. Another conducting polymer that has received considerable attention is polyaniline, which offers advantages such as low cost, environmental stability, and the presence of multiple functional groups that can coordinate with metal ions and dyes [76,77]. Furthermore, due to its cationic nature, polyaniline undergoes protonation, enabling the adsorption of anionic dyes to its amino groups. However, modification of surface charge can be conducted to accommodate cationic dyes as well [78]. Additionally, polyimide provides significant thermal stability, chemical and radiation resistance, and the capacity to be processed into films, fibers, coatings, or composites. These properties are essential for this polymer in extreme environments [79,80].

**Table 3:** Overview of polymeric nanomaterials for water treatment.

Type of Polymeric Nanomaterial	Mechanism of Pollutant Capture	Advantages	Limitations	Refs.
Chitosan	Chemical–physical adsorption: Colloids have been removed by the coagulation and flocculation procedure	Adsorption of various polluants (inorganic, organic, and even toxins) Unique physicochemical properties Can be functionalized with different nanoparticles Environmentally friendly Properties such as biocompatibility, biodegradability, non-toxicity, and bioactivity make chitosan safe to use	The crystallinity and porosity of polymers vary based on the raw materials and extraction method Requires the grafting of functional groups such as amino (-NH_2_), carboxyl (-COOH), thiol (-SH), and hydroxyl (-OH) to interact with the pollutants, fix the heavy metals, and increase the adsorption capacity	[58,59,60,61,62,64,65]
Cellulose	Chemical bonding: New functional groups can be formed via halogenation, esterification, etherification, amination, sulfonation, acetylation, or with hydrophobic groups Ion exchange: Pollutant ions can be absorbed by the functional groups of cellulose	Characterized by biodegradability and low toxicity It is a renewable source, being the most bountiful polymer worldwide Cost-effective	It is considered an efficient adsorbent only when the pH of the solution is between 3 and 7 Large-scale application is impeded by high treatment costs, and limited durability and reusability	[66,67,68,81,82]
Lignin	Bioremediation: Lignin-based gels or spheres can capture the heavy metal on their surface Photocatalytic degradation: Combined with semiconductors, via a photocatalytic system, lignin has the capacity to promote charge separation, enhance surface area, and create favorable conditions for photocatalytic reactions	Tunable physicochemical properties Can be combined with nanocomposites, blends, carbonaceous materials, porous materials, and semiconductors to enhance the target water treatment application Used as a precursor in the production of carbonaceous adsorbents, which boosts the adsorption of multiple contaminants It is a major constituent of plants and also an abundant residual product of the cellulose and bioethanol industry Cost-effective	For better efficiency, lignin must be modified by cross-linking, grafting, the addition of hydrogel beads, composites, and bio(nano)sorbents Requires advances in technology and controlled environmental conditions Efficacy depends on the choice of techniques for separation/preparation from available raw materials	[66,71,72,83,84]
Polypyrrole	Photothermal water evaporation and photocatalysis: As a π-conjugated polymer, polypyrrole’s superior photo-physical properties enable it to decompose pollutants/generate chemical reactions, or facilitate water evaporation using sunlight	Versatile sorbent Has environmental stability, redox activity, and high affinity for both inorganic and organic contaminants Tunable physicochemical properties Surface area and porosity can be enhanced to capture target molecules.	It is an agglomerated powder with poor charge separation, and it is necessary to assemble it into a compact porous aerogel	[74,75]
Polyaniline	Chemical adsorption: Active groups, imine (-C=NH) and amine (-NH_2_), interact via protonation with molecules of different pollutants	Profitable adsorbent Environmental stability Benefits from simple synthetic approaches and low costs Its different structural shapes make it an excellent adsorbent for toxic metal ions	Due to the amine active group, the pH of the solution is an essential parameter in order to activate binding sites by releasing the protons	[76,77,78]

### 2.4. Other Nanomaterials

Besides carbon-based nanomaterials, metal and metal oxide nanoparticles, and polymeric nanostructured materials, in recent years, other materials have also been studied, gaining interest for water treatment due to their adsorption efficiency for various pollutant categories. The main properties of nanomaterials, such as high surface area, chemical and thermal stability, low density, porosity, and their capacity for functionalization, offer a wide range of possibilities for studying targeted environmental pollutants [17,85,86,87]. For example, functional MOFs have shown good adsorption capacity for heavy metal ions, such as chromium, cadmium, copper, lead, and mercury, and for weakly polar, amphoteric, and ionic organic contaminants, like rhodamine B, methylene blue, methyl orange, Congo red, and indigo carmine [17,88]. Similar to MOFs, magnetic covalent organic frameworks (COFs) have been investigated and shown to have an effective removal capacity for inorganic and organic pollutants, owing to their crystalline porous structure and ordered π structure. Magnetic-silica-based composites effectively remove dye contaminants from contaminated water. They are easy to reproduce and regenerate for many decontamination cycles [17]. Biochar and clay-based composites have also been investigated, resulting in the effective capture and immobilization of heavy metals due to their structural and chemical properties, as well as their cost-effectiveness [89,90].

### 2.5. Case Studies of Nanomaterial Applications in Water Treatment

Case studies from several countries have already implemented nanomaterial-based drinking water systems to offer realistic solutions to challenges associated with drinking water contamination problems (Table 4). These case studies give a clear view of the efficiency of nanomaterials in purifying water from heavy metals, organic pollutants, and pathogens. The value of these technologies would be greater in areas where conventional water treatment methods are inadequate or too costly [91]. Another study showed that this technology provides more efficient and significantly less expensive results in the long run, as water purification systems leverage the enormous surface area and high reactivity of materials, as well as their ability to selectively remove pollutants, ultimately making them scalable for improving water quality [11].

A striking instance comes from India, where arsenic-contaminated groundwater was successfully treated with nanoscale nZVI. In areas such as West Bengal and Bihar, excessive arsenic in drinking water poses a critical health hazard [92]. The integration of nZVI into filter matrices has proven to effectively reduce arsenic levels through adsorption and immobilization, facilitated by redox reactions. This advancement has mitigated the financial burdens faced by remote areas that once depended on centralized water treatment facilities [93]. Additionally, iron oxide nanoparticles (Fe_3_O_4_ NPs) have been utilized in community water filtration systems to address arsenic pollution. Their magnetic characteristics allow for straightforward separation and regeneration, thereby improving their efficacy in ensuring the safety of drinking water [94].

In China, TiO_2_ nanoparticles have been successfully used in photocatalytic systems to clean wastewater from industrial enterprises. An outstanding application of this technology is the decomposition of organic pollutants, particularly dyes produced by textile plants. These conditions compelled TiO_2_ to generate ROS upon exposure to UV light, thereby degrading pollutants into less harmful molecules. This process contributed to mitigating industrial water pollution in the textile sector, which is a significant environmental concern in China [95,96].

Silver nanoparticles (Ag NPs) are used in portable water filtration systems for rural areas of South Africa, where access to clean drinking water is limited. Consequently, these nanoparticles have been integrated into filtration membranes to facilitate the elimination of bacteria and pathogens from drinking water. Thus, this approach provides a cost-effective and effective way to improve water quality when conventional water treatment plans are out of reach, especially in remote areas without access to reliable water treatment facilities [97]. Finally, CNTs are also being used on a pilot scale in the United States to purify municipal wastewater for organic pollutants. CNTs have been combined into filtration systems to attain high adsorption capacity for organic and inorganic contaminants. The pilot projects have demonstrated the capability of CNT-based systems to enhance the efficiency of complex pollutant removal processes, offering a promising alternative to conventional AC filters commonly used in practice [98].

In Switzerland, polystyrene nanoparticles have been successfully used in municipal water treatment plants to remove pharmaceutical residues. These polymeric nanoparticles exhibit selective adsorption of antibiotics and hormones, thereby markedly reducing pharmaceutical contamination in drinking water sources [99]. In Serbia, TiO_2_ nanoparticles have been used in innovative photocatalytic reactors to reduce organic pollutants in water. These nanoparticles utilize UV-activated photocatalysis, confirming efficient pollutant breakdown and benefiting from self-cleaning surfaces that improve efficiency over longer durations [38]. In Germany, these nanomaterials have been incorporated into water filtration membranes to achieve ultrafiltration levels of contaminants. Due to their high mechanical strength and significant adsorption capacity, these membranes effectively remove organic and inorganic pollutants [100]. In South Korea, ZnO nanoparticles have been combined into antimicrobial water filtration membranes for rural communities. The antibacterial properties of these agents inhibit microbial proliferation, thereby improving water disinfection and providing an economical solution for decentralized water treatment systems [37]. Nanocellulose-based membranes have also been developed in Malaysia for desalination and microplastic removal from water sources. As a renewable and biodegradable material, nanocellulose membranes provide a eco-friendly approach to water purification [101,102].

**Table 4:** Overview of case studies of nanomaterial applications in water treatment.

Country	Nanomaterial	Application	Outcome	Refs.
India	nZVI; Fe_3_O_4_	Arsenic removal from groundwater Deployed in community water filtration systems for arsenic removal	Significant reduction of arsenic levels in drinking water Magnetic properties allow for easy separation and regeneration, improving drinking water safety	[92,93,94]
China	TiO_2_	Photocatalytic degradation of industrial wastewater	Efficient removal of organic dyes from textile wastewater	[95,96]
South Africa	Ag NPs	Pathogen removal in rural filtration systems	Improved microbial water quality in rural communities	[97]
United States	CNTs	Removal of organic pollutants from municipal wastewater	Enhanced removal of pharmaceuticals and pesticides	[98]
Switzerland	Polystyrene NPs	Used in municipal water treatment plants to remove pharmaceutical residues	Selective adsorption of antibiotics and hormones, reducing pharmaceutical pollution in drinking water	[99]
Serbia	TiO_2_ NPs	Utilized in advanced photocatalytic reactors for the degradation of organic pollutants	UV-activated degradation of pollutants and self-cleaning surfaces enhance efficiency	[38]
Germany	Graphene-Based Nanomaterials	Implemented in water filtration membranes for the ultrafiltration of contaminants	High mechanical strength, exceptional adsorption efficiency for heavy metals and organic pollutants	[100]
South Korea	ZnO NPs	Integrated into antimicrobial water filtration membranes for rural communities	Inhibits bacterial growth, enhances water disinfection, and is cost-effective for decentralized systems	[37]
Malaysia	Nanocellulose-Based Membranes	Developed for desalination and the removal of microplastics from water	Renewable, biodegradable, and highly efficient in filtering contaminants	[101,102]

Abbreviations: NPs—nanoparticles.

## 3. Fate and Behavior of Nanomaterials in the Environment

### 3.1. Transport and Mobility of Nanoparticles in Water Bodies

Nanoparticles have emerged as a subject of extensive research owing to their distinctive properties, which make them suitable for a diverse array of applications, including water purification, medical treatments, and various industrial processes. These concerns involve several environmental effects, mainly transport and mobility in aquatic ecosystems. For some time, once these particles are introduced into the water, they may undergo complex fates due to dispersion, aggregation, sedimentation, and dissolution. Understanding the behavior of nanosystems in aquatic environments is critical for evaluating their environmental risks and developing control strategies [2,31].

The summary of the fate and behavior of nanomaterials in the environment is shown in Table 5.

#### 3.1.1. Mechanisms of Dispersion, Aggregation, and Sedimentation

Nanoparticle dispersion is the phenomenon in which nanoparticles become uniformly distributed throughout a water body. Factors affecting this process include the size and shape of the nanoparticle, surface charge dynamics, and hydrodynamic factors such as turbulence, temperature, and, at times, the presence of other chemicals in the water column [31]. Because smaller nanoparticles have relatively low settling velocities, they tend to remain suspended in the water column for longer periods, allowing them to disperse over a larger area. Conversely, larger particles or agglomerated nanoparticles settle more quickly and more readily under the influence of gravity. The way nanoparticles disperse also fundamentally governs their potential reactivity with organisms or other components of the aquatic environment [103].

Aggregation, therefore, is yet another significant process by which nanoparticles will feel each other and move in the aquatic environment. When nanoparticles aggregate, they come together and eventually form larger particles. In fact, aggregation may dramatically decrease nanoparticles’ mobility due to larger agglomerates, which are better candidates for loss through sedimentation as they settle out of the water column. The degree of aggregation depends on the surface properties of the nanoparticles, such as surface charge and hydrophobicity, and the water’s chemical composition. Highly charged nanoparticles repel each other and, therefore, will remain dispersed. Neutral or weakly charged nanoparticles will aggregate even more easily [104,105].

Sedimentation is the settling of nanoparticles out of the water column to accumulate in sediments at the bottom of the water body. Sedimentation rates are highly dependent on nanoparticle size and density, as well as on nanoparticle aggregation. Generally, larger and denser particles, as well as aggregated nanoparticles, settle faster than smaller and less dense particles. Sedimentation is thus of prime importance in determining the ultimate long-term fate of nanoparticles within aquatic systems. Once in the sediments, nanoparticles may either be buried or resuspended under specific environmental conditions, such as storm events or disturbances [106,107].

#### 3.1.2. Aggregation and Dissolution of Nanoparticles

Aggregation and dissolution greatly influence the stability and transport of particles in aquatic systems. Particle aggregation is strongly influenced by the environmental conditions surrounding them. These factors include pH, ionic strength, and NOM in the water. pH greatly affects the surface charge of nanoparticles and, hence, their stability or tendency to aggregate. Most nanoparticles possess surface charges mainly because of the ionization of functional groups at their surfaces. At low pH, which corresponds to acidic conditions, negatively charged surface nanoparticles can be protonated. As a result, electrostatic repulsion will decrease, and hence aggregation will be facilitated. Conversely, at high or low pH values, the negative charge on the particle surface increases, thereby further stabilizing the particles and decreasing aggregation. The isoelectric point of the particles, where they carry no net surface charge, is the point of maximal aggregation [105,114].

Basically, the ionic strength of the water is the amount of dissolved salts within the water. The higher the ionic strength, the more it compresses the electrical double layer around the nanoparticles, thereby decreasing the repulsion between them and leading to their aggregation when they come into very close proximity [115]. For example, suppose the water is salty or hard (in which case the water has high concentrations of divalent cations such as calcium or magnesium). In that case, such chemicals significantly increase the probability of nanoparticle aggregation compared to fresh water, which has low ionic strength. These divalent cations are particularly those that neutralize the nanoparticles’ surface charge; hence, they lead to even faster aggregation [105,108].

Humic acids and fulvic acids, along with other organic substances, can be adsorbed onto nanoparticle surfaces and affect their stability [112,116]. It can work both ways, either stabilizing or destabilizing them, depending on the concentration and nature of NOM compounds. Under certain conditions, it creates steric hindrance, leading to increased aggregation and, thus, the stabilization of nanoparticles in suspension. This is known as steric stabilization. However, under other conditions, it bridges two nanoparticles, facilitating their aggregation due to its tendency to agglomerate. The role is specific in the type of nanoparticles and organic matter within the water [105,108].

### 3.2. Interaction with Natural Organic Matter

Humic substances, fulvic acids, and other organic compounds constitute the general aspect of natural organic matter (NOM) in aquatic systems. The behavior, fate, and efficiency of nanomaterials in pollutant removal are, in most cases, mainly determined by their interactions with these organic compounds. In adsorption, complexation, and surface coating, NOM interacts with nanomaterials through several mechanisms, including participation, which can influence nanomaterial stability and their ability to remove contaminants from water [116].

Complexation is one of the primary mechanisms by which NOM alters nanomaterials. Reactive nanomaterials can be complexed with NOM to form stable complexes. Such complexes dramatically alter the surface characteristics of nanomaterials, including surface charge and hydrophobicity. For example, in metal nanoparticles, NOM will also adsorb to the surface but may form a coating that alters particle stability [109,110]. Such a coating can provide nanoparticles with colloidally stability by providing steric protection —electrostatic repulsion that maintains them in suspension in the aqueous phase for extended periods. On the other hand, NOM can act as a flocculant, binding nanoparticles together and increasing their tendency to aggregate and thus sediment from the suspension. Reaction kinetics will largely depend on the concentration and composition of NOM, as well as on the geochemistry of the nanoparticle surface [108,109].

The effect of NOM on pollutant removal is very complex and strongly dependent on the type of nanomaterial and the contaminant. In some cases, NOM likely enhances pollutant removal efficiency [109]. For example, TiO_2_ can enhance the photocatalytic degradation of organic pollutants by providing more reactive sites for adsorption–desorption or simply by preventing pollutants from settling on the nanomaterial surface [38]. NOM can help entrap hydrophobic organic pollutants, facilitating their interaction with the nanomaterial and enhancing removal efficiency. In other systems, NOM interactions with nanomaterials reduce pollutant removal efficiency [82]. For example, carbon-based nanomaterials, such as GO and CNTs, lead to a competition between the NOM and pollutants for adsorption sites on the surface of the nanomaterial. When competition is rife, there will be fewer sites where the contaminants could be adsorbed; hence, the overall removal efficiency will be reduced [24,32,98]. Another way the NOM coverage of nanomaterials affects the process is by blocking active sites, thereby preventing direct interaction between nanomaterials and pollutants, particularly when the removal process depends on surface reactions such as adsorption or photocatalysis. In addition, NOM also governs the environmental fate and transformation of nanomaterials, as environmental transformations are considered to profoundly change reactivity, stability, and toxicity of nanomaterials; they will be discussed in more detail in the next section [40,99].

### 3.3. Environmental Transformation (Oxidation, Sulfidation, Etc.)

Nanomaterials introduced into the environment undergo a series of chemical and physical transformations, leading to some degree of property changes. Such transformations, such as oxidation and sulfidation, are essential for altering the reactivity, toxicity, and overall behavior of nanomaterials in the environment [36,113]. Therefore, knowledge of these transformations is important for predicting the long-term environmental impact of nanomaterials. Oxidation is probably the most common type of environmental change, especially for Ag NPs and nZVI-based nanomaterials. It is known that surface oxidation of metal nanoparticles occurs under oxidizing conditions, leading to the formation of oxide layers on metal nanoparticles and, thus, increasing their chemical reactivity and, consequently, their toxicity [35,36]. For example, the oxidation of silver nanoparticles to silver ions (Ag^+^) is well-known and increases toxicity compared to the nanoparticulate form. This process increases the potential risks posed by nanomaterials to aquatic organisms because the release of metal ions can cause harmful effects. Nanoparticles are passivated via oxidation, which forms an oxide shell that decreases reactivity and, ultimately, the efficiency of pollutant interactions in water treatment applications [111].

Another crucial process is sulfidation, in which metal nanomaterials come into contact with sulfur compounds, primarily sulfides, in sediments or wastewater. It can significantly alter nanomaterial properties because sulfidation forms metal sulfides, which are usually less soluble and less toxic than their metal oxides or metallic forms [112]. For instance, silver nanoparticles will change into much less toxic, more stable silver sulfide (Ag_2_S). This would drastically reduce the release of toxic silver ions into the environment, thereby greatly reducing environmental risks. Sulfidation can also reduce the performance of nanomaterials in water treatment because it alters their oxidative (catalytic or adsorptive) activity in sulfide form. For example, in the case of nZVI, it would reduce the nanoparticles’ ability to degrade organic contaminants or immobilize heavy metals, making it effective for remediation [36,57,112].

Dissolution is very crucial, mainly for the metal oxide nanoparticles of ZnO and TiO_2_. Nanoparticles dissolve in the aqueous phase and release metal ions in the water. The rate of dissolution is governed by the abiotic factors, among which the most important are pH, ionic strength, and the presence of NOM. For example, ZnO would dissolve more quickly at lower pH values, thus making zinc ions (Zn^2+^) toxic to aquatic organisms at concentrations above EC50 [37,38,43]. Nanomaterial dissolution increases bioavailability and, hence, toxicity in aquatic life. Sometimes, the reactivity of nanomaterials decreases after dissolution, since they are in nanoparticulate form and become ions once dissolved; this is the difference in chemistry [43,54,83].

In addition to oxidation, sulfidation, and dissolution, nanomaterials can also undergo other environmental transformations such as surface functionalization and photoinduced transformations [36,113]. An example of metal oxide nanoparticles is TiO_2_, known for its photocatalytic properties; moreover, these nanomaterials can be used to generate ROS in the presence of UV light. Sunlight in a natural aquatic environment can induce photochemical reactions that can alter the surface properties of nanomaterials, thereby changing their reactivity and toxicity. Under certain conditions and with specific nanomaterial properties, photochemical transformations can improve or decrease the removal efficiency [95,113].

## 4. Toxicological Effects on Aquatic and Terrestrial Ecosystems

### 4.1. Nanomaterial-Induced Toxicity in Aquatic Organisms

Nanomaterials have wide applications in industry, medicine, and environmental biotechnology because of their unusual physicochemical properties resulting from extremely small size, high surface area, and reactivity. However, these properties that indicate elevated reactivity may also raise concerns about the toxicity of these materials to aquatic ecosystems (Table 6). After entering the aquatic environment, nanomaterials come into contact with diverse groups of aquatic biota, which, in turn, may lead to toxic effects at different levels of the trophic chain, ranging from fish to crustaceans, algae, and other aquatic species [108,109].

#### 4.1.1. Impacts on Fish

Fish generally occupy the highest trophic levels among aquatic organisms. Moreover, they are highly sensitive to nanomaterial-induced toxicity because they absorb it from water and sediments and are ultimately incorporated into contaminated food sources. Some metal nanoparticles identified as inducing oxidative stress, gill damage, and inflammation in fish include Ag NPs and ZnO. For example, silver nanoparticles may produce ROS, which, in turn, will cause oxidative damage in fish gills, liver, and other tissues. This oxidative stress can lead to lipid peroxidation, DNA damage, and apoptosis, with several fundamental consequences, eventually impairing respiration, reproduction, and growth [16,108,111]. Other TiO_2_ NPs are widely used in sunscreen and water treatment formulations and have been shown to induce toxic gill tissue damage and alter fish blood parameters, thereby compromising the general condition and survival of fish populations [16,24,96].

#### 4.1.2. Impacts on Crustaceans

Crustaceans, including shrimp, crabs, and lobsters, are vital to the aquatic ecosystem, as they aid in nutrient cycling and serve as a significant food source for organisms at higher trophic levels. Nevertheless, nanomaterials may exert toxic effects on the physiology and behavior of these crustaceans. Previous studies reported that the uptake of carbonaceous nanomaterials, particularly GO and CNTs, by crustacean digestive systems leads to reduced feeding efficiency, reduced mobility, and increased mortality [43,108]. Other observations include the fact that metal-based nanoparticles, for instance, Cu NPs, initiate retarded development in crustaceans by causing delayed molting and inhibited growth. Generally, the toxicity of nanomaterials toward crustaceans arises from ROS generation, leading to oxidative damage to cellular structures, as well as disruption of energy metabolism and immunologic impairment [22,117].

#### 4.1.3. Impacts on Algae

Primary producers in aquatic ecosystems include algae, as they immensely contribute to the food web and water quality. Nanomaterials can inhibit algal growth, photosynthesis, and reproduction, thereby establishing and maintaining ecosystem imbalances. For example, nanoparticles of metal oxides, including ZnO and TiO_2_, can significantly enhance ROS production and damage the photosynthetic apparatus of algae [40,43,117]. Consequently, this results in lowered chlorophyll synthesis, suppressed photosynthesis, and diminished growth rates in algal populations. Additionally, carbon-based nanomaterials, such as graphene oxide, can absorb light, thereby inhibiting algal photosynthesis. When toxic, the effects will be transmitted not only through the algae but also downstream to herbivores and higher levels of the food web, which directly depend on algae as a food source [118].

#### 4.1.4. Impacts on Other Aquatic Life

Various aquatic organisms, such as bivalves, amphibians, and microorganisms, exhibit responses to the toxicity associated with nanomaterials. In bivalves such as mussels and clams, the introduction of nanomaterials results in bioaccumulation in soft tissues via particulate-feeding pathways, which subsequently causes oxidative stress, inflammation, and a decline in filtration efficiency [117,119]. Additionally, amphibian larvae, characterized by their permeable skin, demonstrate heightened sensitivity to toxic nanomaterials found in their aquatic environment. Nanomaterials can arrest amphibian embryonic development, leading to malformations, reduced growth, and altered behavior. In the aquatic system, bacteria and protozoa, which are critical for nutrient cycling and decomposition of organic matter, are developing normally. The nanomaterials may exhibit inhibitory effects on these microbial groups, thereby not only diminishing their growth rates but also altering their composition and metabolism, which could have long-term implications for ecosystem function [120].

**Table 6:** Summary of nanomaterial-induced toxicity in aquatic organisms.

Organism Group	Observed Effects	Mechanism of Toxicity	Representative Nanomaterials	Refs.
Fish	Gill tissue damage Oxidative stress in the liver and other organs Lipid peroxidation, DNA damage Apoptosis leading to reduced respiration, reproduction, and growth Altered blood parameters and general physiological dysfunction	Generation of ROS Accumulation in tissues via water, sediment, and food Cellular damage through oxidative mechanisms	Ag NPs, ZnO NPs, TiO_2_ NPs	[16,24,96,108,111]
Crustaceans	Reduced feeding efficiency Diminished mobility and increased mortality Retarded development and delayed molting Inhibited growth and behavioral impairment	Ingestion and accumulation in the digestive tract ROS-induced damage to cellular structures Disruption of energy metabolism and immune response	GO, CNTs, Cu NPs	[22,43,108,117]
Algae	Growth inhibition Disruption of photosynthesis and chlorophyll production Impaired reproduction and ecological imbalance	ROS generation damages photosynthetic machinery Physical blockage of light absorption Disruption of cell signaling and energy pathways	ZnO NPs, TiO_2_ NPs, GO	[40,43,117,118]
Other Aquatic Life (e.g., bivalves, amphibians, microbes)	Bioaccumulation in soft tissues (e.g., mussels) Inflammation and oxidative stress Reduced filtration and metabolic function Developmental arrest and malformations in amphibians Altered microbial community structure and nutrient cycling	Uptake through particulate-feeding or permeable skin ROS-mediated cellular damage Interference with microbial composition and metabolism	Ag NPs, TiO_2_ NPs, various metal-, and carbon-based NPs	[117,119,120]

### 4.2. Bioaccumulation and Biomagnification

The principal matter regarding the release of nanomaterials into the aquatic ecosystem concerns their potential for bioaccumulation and biomagnification. In essence, bioaccumulation is the accumulation of nanomaterials in organisms’ tissues over time, whereas biomagnification is an increase in their concentration as they move up the food chain [35,43,109].

#### 4.2.1. Bioaccumulation in Aquatic Organisms

**Figure 2:**
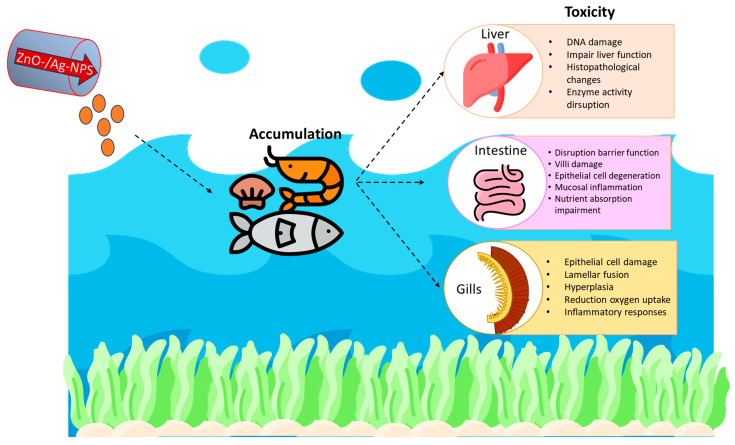
Toxic effects of nanomaterials in aquatic organisms. Created with elements from Flaticon Basic License CC3.0 (Creative Commons).

In aquatic animals, the major pathways by which nanomaterials such as Ag NPs, ZnO, and Cd-QDs are delivered into tissues include uptake via water, ingestion of contaminated food, or contact with sediments [35,108]. Upon their introduction into fish, crustaceans, and mollusks, these nanoparticles have the potential to be disseminated to multiple organs, including the liver, gills, and kidneys, where they may also cause toxic effects [16,108,111] (Figure 2). For instance, silver nanoparticles accumulate in the gills, liver, and intestines of fish over time, leading to oxidative stress, inflammation, and organ dysfunction. Similar studies have also found that ZnO NPs can bioaccumulate in crustacean and molluscan tissues, causing developmental retardation and reduced survival [119,120]. The bioaccumulation of nanomaterials in aquatic animals ensures longevity and health risks, yet more risk arises because it is a potential medium through which such materials may gain access to the human body through the consumption of contaminated seafood [16,55].

#### 4.2.2. Biomagnification in Aquatic Food Chains

Biomagnification refers to the process by which nanomaterials accumulate in concentration as they move from lower trophic levels to higher trophic levels. Through predation, nanomaterials that bioaccumulate in the tissues of lower trophic invertebrates and plankton can be transferred to fish and avian species. When these organisms serve as prey in the food chain, the concentration of nanomaterials increases to potentially dangerous levels in the top predator, sometimes humans [16,109]. For instance, the aquatic food chain showed that silver nanoparticles were biomagnified in predatory fish more than in non-predatory fish. Likewise, aquatic ecosystems have been demonstrated to biomagnify cadmium-based quantum dots, with higher concentrations found in fish that consume invertebrates exposed to the pollution sources. Concerns about human health implications arise from the biomagnification of nanomaterials in aquatic food chains; humans, as top predators in most cases and consumers of contaminated seafood, may be exposed to higher concentrations of nanomaterials [109,118].

### 4.3. Oxidative Stress and Cellular Damage Mechanisms

Due to their small size and high surface area, nanomaterials can generate ROS upon exposure to aquatic environments. ROS can exist in the form of free radicals, such as the superoxide anion (O_2_^−^) and the hydroxyl radical (OH^−^), as well as non-radical species, such as hydrogen peroxide (H_2_O_2_); yet, all are highly reactive and cause oxidative damage to biological components [119,121]. Once inside aquatic organisms, nanomaterials react with their cells to initiate what may be termed oxidative stress, which is an imbalance between ROS production and the biological system’s power to clear up the reactive intermediates [24,54].

ROS generation by nanomaterials mainly involves their surface chemistry. Here, Ag, ZnO, and TiO_2_ catalyze the reactions under certain conditions, i.e., when exposed to light [95,108,111]. For example, TiO_2_ nanoparticles exhibit photocatalytic activity in UV light, which causes oxidative damage in the surrounding cells. This damage is manifested by peroxidation of cellular membrane lipids, denaturation or inactivation of proteins, or mutations or fragmentation of nucleic acids, including DNA. Such molecular disruptions disrupt normal cellular function and can be fatal when oxidative stress is strong and sustained [111].

In fish and other aquatic organisms, sensitive tissues where oxidative stress can cause oxidative inflammation and tissue degeneration, leading to impaired organ function, include the gills, liver, and kidneys, where ROS-induced damage has been described. In the gills, ROS generation triggers apoptosis and necrosis, as well as the formation of chronic respiratory dysfunction and reduced survivability. It comes with immunosuppression on the side that makes organisms extra susceptible to diseases. Therefore, ROS production by nanomaterials is a major pathway through which these materials exhibit toxicity toward aquatic organisms [111,117,118].

### 4.4. Effects on Microbial Communities and Biogeochemical Cycles

While larger aquatic biota are expected to experience some effects, a much higher threat level is directed towards microbial communities, which are “lifelines” for ecosystem health and for maintaining and driving the various biogeochemical cycles. Bacteria, archaea, and fungi play key roles in nutrient cycling, organic matter decomposition, and chemical transformation of substances within aquatic environments [122]. However, the introduction of nanomaterials into such systems definitely disrupts the proper microbial community and the control of critical processes. A major mode by which nanomaterials impact microbial communities is through their antimicrobial properties. Perhaps metal-based nanoparticles, such as Ag and CuO, play a significant role. These nanomaterials will likely interact with the microbe’s cell membrane, physically destroying it, or hampering various important biological processes via metal–ion-mediated entry. For example, bacterial wall integrity is compromised by AgNPs, leading to gradual leakage of cellular contents and, therefore, probable cell death. This kind of disinfection action has some considerations regarding the unintended consequences of applied water disinfection actions on beneficial bacteria in aquatic environments [119,121]. Changes in nutrient cycling of the nitrogen and carbon cycles would be support-dependent on system stability due to the fact that bacteria would be dependent on nitrogen fixation or nitrification (conversion of ammonia into nitrate), which would be inhibited by the toxic nanomaterials. Some bacteria may be involved in nitrogen fixation or nitrification, which may be inhibited following exposure to toxic nanomaterials; as a result, fewer nutrients are made available to primary producers such as algae. Nanomaterials cause their effects on microbial decomposition because they change the decomposer bacteria’s and fungi’s activities, hence resulting in a possible retardation of organic matter breakdown with implications for the carbon cycle [118,122].

Table 7 summarizes toxicological, environmental fate, and ecological impact aspects in the comparative assessment of polymeric nanomaterials with other nanomaterials. Polymeric nanomaterials, for example, poly (lactic-co-glycolic acid) (PLGA), typically have much lower intrinsic toxicity than metal, metal oxide, or carbon-based nanomaterials. They are highly biocompatible, with the possibility of controlled degradation; hence, they are very attractive for environmental applications; however, toxicity may emanate from specific surface modifications or residual monomers [83]. On the other hand, more metal-like Ag, TiO_2_, and ZnO NPs display higher apparent toxicities because they release metallic ions and also generate ROS, which damage cellular structures and lead to bioaccumulation in aquatic organisms [43,108]. Carbon-based nanomaterials, including CNTs, show variable toxicity profiles determined by specific chemical and morphological properties but can cause oxidative stress and inflammation [117]. Most polymeric nanomaterials are made from biodegradable polymers. They break down into harmless by-products and do not persist in the environment for long periods. Only a few non-biodegradable types could break down into micro- and nanoplastics, which might persist in the aquatic environment and pose potential hazards to organisms [117]. Metal or metal oxide can either persist or aggregate within the environment, in addition to undergoing chemical transformation inside the environment itself. The possibility that they may release toxic ions that will accumulate within sediments or biota gives rise to concerns regarding their long-term ecological impacts [108]. Most carbon-based nanomaterials degrade even more reluctantly; their fate depends largely on interactions with NOM and on whether they tend more towards aggregation/settling than to remaining suspended.

Ecologically, the risk is considered to be very minimal from biodegradable polymeric nanomaterials. In fact, incomplete degradation or formation of microplastics disrupts feeding mechanisms and causes physical harm to aquatic life, as reported by Wang et al. [109]. Gao et al. [118] observed that higher reactivity and persistence of metal and metal oxide nanomaterials adversely affect the microbial community, inhibiting algal growth and disrupting food webs through bioaccumulation and toxicity. Carbon-based nanomaterials may also have negative impacts on a wide range of organisms, affecting their growth and reproduction, and may interact with other environmental pollutants, thereby enhancing ecological risks [118].

Carbon-based, metal/metal oxide, and polymeric nanomaterials have a high degree of decontamination that is achieved through either direct interaction, catalytic activity, or specific association. Their environmental risks differ, including toxicity, metal deoxygenation, and persistence, which must be carefully considered. Reusability and recyclability are often impaired by aggregation, structural inefficiency, or energy-intensive recovery methods. Effective design and efficient use of these nanostructures require balancing the process’s effectiveness with safety and potential applications in real-world water treatment settings. High efficiency is not necessarily compromised in the pursuit of environmental sustainability or the potential for multiple applications in real-world water treatment situations [24,43,58].

**Table 7:** Comparative overview of nanomaterials utilized in water decontamination based on their toxicity, environmental fate, and ecological impacts.

Nanomaterials	Toxicity	Environmental Fate	Ecological Impacts	Refs.
**Carbon-based nanomaterials** Example: CNTs, graphene, fullerenes	Toxicity varies widely with structure and purity Long, rigid CNTs can cause asbestos-like effects in organisms Can accumulate in tissues and cause inflammation Often no biodegradable	Extremely persistent in the environment Resistant to biological and chemical degradation Can adsorb pollutants and transport them through ecosystems	Can bioaccumulate in lower organisms Physical damage to cells and tissues Long-term ecosystem impacts are still uncertain but concerning	[16,24,96,108,111]
**Metal/Metal Oxide nanomaterials** Example: Ag, TiO_2_, ZnO	Higher toxicity potential, even at low concentrations Can release toxic metal ions (e.g., Ag+, Zn^2+^) Generate ROS, leading to oxidative stress Known to affect microorganisms, algae, fish, and invertebrates	Do not degrade chemically Transform via dissolution, aggregation, or surface reactions Can accumulate in sediments and biofilms Long environmental residence times	Strong effects on the following: - Algae (growth inhibition) - Microbial communities - Fish development and behavior Can disrupt food webs at low trophic levels	[22,43,108,117]
**Polymeric nanomaterials** Example: PLGA, PEG nanoparticles, Chitosan, polymeric nanoplastics	Generally, lower intrinsic toxicity than metal or metal oxide nanoparticles Often designed to be biocompatible and biodegradable, especially in medical or environmental applications Toxicity depends strongly on the following: - Polymer type (natural vs. synthetic) - Molecular weight - Surface charge (cationic polymers tend to be more toxic) Can still cause cell membrane disruption or inflammation at high concentrations	May degrade over time (especially biodegradable polymers) Breakdown products can be less harmful, but micro- and nanoplastics are a concern Can persist in soil and water if not biodegradable Tend to aggregate, reducing mobility in some environments	Typically, lower acute toxicity to aquatic organisms Chronic effects are less well-understood Potential impacts include the following: - Reduced feeding efficiency in plankton - Bioaccumulation of nanoplastics Risk increases if the particle persists or carries adsorbed pollutants	[58,65,83]

Abbreviations: PEG—polyethylene glycol; PLGA—poly (lactic-co-glycolic acid).

## 5. Knowledge Gaps in Nanomaterial Toxicity Studies

### 5.1. Short-Term vs. Long-Term Exposure Studies

Most toxicity studies on nanomaterials have been based on short-term exposure, typically at very high concentrations of nanomaterials for a short duration. Such studies shed light on immediate acute effects, such as oxidative stress, cell membrane disruption, and mortality in aquatic organisms [121,123]. An illustrative example is silver nanoparticles, which cause severe oxidative damage in both fish and invertebrates under short-term exposure. While these results are quite useful, they do not reflect the actual environmental conditions in which the biota would normally be exposed to comparatively lower concentrations of nanomaterials over long durations [111].

Long-term exposure studies are relatively rare, particularly for the effects of chronic, low-dose exposure to nanomaterials on organisms [124]. Such studies are very important for predicting the effects of long-term exposure to nanomaterials, such as possible bioaccumulation and chronic toxicity [16,111]. For example, a study on zinc oxide nanoparticles has proved that long-term exposure at environmentally relevant concentrations can adversely affect reproductive performance in aquatic organisms; hence, many similar experiments are required [35,108]. Such long-term data gaps on the chronic or delayed sublethal effects of growth impairment and reproductive failure underscore the shortfalls in developing long-lasting transformations of nanomaterials within situational conditions and in their interactions with other environmental components [119].

### 5.2. Lack of Standardized Testing Protocols

A critical problem with studies on the toxicity of nanomaterials is the lack of any standard testing protocols. The distinct core/shell nanoparticle properties themselves can greatly influence toxicity. For example, a smaller particle size would lead to a relatively more exposed surface area and hence more chemical toxicity [125]. The variation and diversity in the properties of nanomaterials allow different studies to have different applied methodologies. The results of provocations may, therefore, carry in some instances inconsistencies and, at times, even contradictions.

The test methods used were developed for testing conventional chemicals and often do not apply to nanomaterials. For example, the dissolution and aggregation of nanoparticles and their interactions with natural organic matter may alter their behavior and toxicity, undermining the effectiveness of standard test conditions against them. Flux towards harmonizing test protocols based on rules for the very different physics of nanomaterials is required [105,108]. Such rules shall include guidance on characterization aspects, such as particle size distributions and surface area measurements of nanomaterials, and the conditions under which testing will take place, reflecting realistic environmental conditions, including pH, temperature, and ionic strength. Standardized methodologies would definitely improve comparative reliability between works and the general understanding of the toxicities of nanomaterials [125].

### 5.3. Challenges in Simulating Realistic Environmental Conditions

One of the most important problems in nanomaterial toxicity is the inability to replicate realistic environmental conditions in laboratory settings. In fact, laboratory experiments are based on rather well-controlled conditions that, by far, do not reflect the complexity and variability of natural environments [126]. To mention how much these factors might influence the behavior and toxicity of nanomaterials, including fluctuating temperatures, the presence of many other organic and inorganic compounds, as well as interactions with other pollutants, has to be mentioned. Such factors very predictably influence the behavior and toxicity of nanomaterials [16]. In natural environments, nanoparticles can undergo transformations that can sometimes reduce their toxicity, such as aggregation, to form larger particles with lower reactivity or dissolve into metal ions—some toxic—thereby increasing their toxicity [24,55]. These changes are very dynamic and hard to replicate in a controlled laboratory environment, where only a fraction of these interactions can be modeled accurately. This necessitates research initiatives focused on conditions that are significantly more dynamic and variable, in comparison to the complexities of real-life ecosystems in which liquid systems operate—considering factors such as organic matter, light exposure, and various other environmental influences arising from all conceivable sources [101,108].

### 5.4. Uncertainty in the Fate of Transformation Products

Nanomaterials may transform chemically or physically while in the environment, for example, through oxidation, sulfidation, or dissolution; these transformations usually greatly alter their properties and toxicity [118]. A metal-based example is silver or zinc oxide nanoparticles, which dissolve into metal ions, which are normally more toxic than the nanoparticles themselves. Alternatively, the reactivity of the metal-based nanoparticles would be enhanced or reduced by oxidation or sulfidation, depending on environmental conditions. Although this is of primary interest for understanding the fate and toxicity of transformed nanomaterials, relatively few studies have been conducted on these transformations [126,127]. The toxicity profiles of the transformation products compared to the original nanomaterials might articulate completely different changes in properties that, in turn, could result in unexpected risks, both to the environment and health [16,24]. For example, although the toxicity of silver nanoparticles transformed in natural waters into Ag_2_S is low for aquatic organisms, substantial further research must be conducted to reveal how these transformations respond differently to varied environmental conditions. Therefore, environmental researchers need to urgently bridge this wide gap in information and understanding [128].

## 6. Risk Assessment and Regulatory Frameworks

The increase in the use of nanomaterials in industrial and other activities, such as water treatment, is evident in the general concern about their risks to human health and environmental safety. The imperative is development, as things scale up, and the application of nanomaterials requires robust risk assessment models and the effective implementation of related regulatory frameworks to ensure safe use and sustainable application. While some available guidelines are already in place and can provide a first view of the safety issues related to nanomaterials, such matter-of-fact-bound regulatory frameworks do not work well because the inherent characteristics of nanomaterials pose specific challenges [92,110].

### 6.1. Existing Guidelines for Nanomaterial Safety

A number of regulatory bodies based in different countries have, in various ways, acknowledged the need to manage risks associated with nanomaterials. The Environmental Protection Agency (EPA) has issued guidance on engineered nanomaterials under the Toxic Substances Control Act (TSCA) in the US. Under TSCA, EPA has required reporting of the composition of the nanomaterial, potential human exposure, and environmental effects. These regulations essentially treat nanomaterials as ordinary chemicals; many feel they fail to adequately account for the special characteristics of nanoscale materials, such as their surface reactivity, large surface area, and ability to traverse biological barriers [129]. In the European Union, the control of nanomaterials is handled within the REACH (Registration, Evaluation, Authorization, and Restriction of Chemicals) framework, which is designed to ensure a high level of protection of human health and the environment from the risks that chemicals can pose. REACH introduced specific provisions under which companies would have to provide more detailed data on the properties and behavior of nanomaterials. Those provisions take into account issues such as particle size distributions and nanomaterial solubility and require much more specific data than for the corresponding bulk chemicals. The major drawback, however, remains the large diversity of nanomaterials and the very incomplete knowledge of their long-term effects. Although these guidelines advance in nanomaterial safety regulation, few of them cover this area comprehensively. Many regions face this gap concerning the exponential pace of innovation in nanomaterials, and it creates an imbalance between the growing industrial applications and research [130].

### 6.2. Emerging Regulations for Water Treatment Applications

One of the most promising applications of nanomaterials is in the treatment of water and the ensuing decontamination. A high efficiency factor has been observed in CNTs, followed by graphene oxide and metal nanoparticles, in removing pollutants, including heavy metals, organic contaminants, and pathogens [28,81]. The very features that prove them to be so successful in treating water present some problems when it comes to their efficacy: their small size and their reactivity amount to concerns involving environmental and health risks when not properly regulated. No specific regulations today apply to the use of nanomaterials in water treatment. Thus, there is an urgent need for clear regulatory guidelines on how these materials may be safely incorporated into water treatment systems, particularly for their disposal as used nanomaterials, which may accumulate in natural water bodies or drinking water systems. Nanomaterials breach aquatic ecosystems, posing possible risks to marine life, bioaccumulation in organisms, and the formation of potentially harmful by-products [43,129,131].

To address these concerns, nanomaterials applied towards decontaminating water demand guidelines to be structured urgently and should confine their boundaries. These rules and regulations should specify the amounts that may be applied, how the environment will be checked, and the disposal conditions. Regulations are expected to be formed, taking into account the entire life cycle of nanomaterials in water treatment systems, from production up to probable environmental release, to reduce the hazards. With such specialized regulations in place, government authorities will help achieve the benefits of nanotechnology in water treatment while always keeping the environment and human health safe [100,130].

### 6.3. Risk Assessment Models for Environmental Impact

Risk assessment models measure the environmental impact and are often not appropriate for traditional use since the behavior of nanomaterials differs significantly in environmental systems. For example, a risk assessment model is incomplete until aggregation, dissolution, and compound interactions are considered, because these are behavior aspects of nanomaterials that are far above those of bulk chemicals, change toxicity, and environmental persistence [105,132]. Thus, a complete risk assessment of a nanomaterial should involve modeling of its fate and transport, specifically how it moves through the atmosphere, hydrosphere, and pedosphere. These models should consider the particle size, surface charge, and environmental parameters, such as pH and ionic strength, that affect nanomaterial behavior. The consideration of bioaccumulation and biomagnification potential, that is, the accumulation of nanomaterials in organisms and an increase in the concentration of the nanomaterials up the food chain, respectively, should form one of the primary considerations in risk assessment, more especially for any application associated with water [35,43].

### 6.4. Future Directions for Policy Development

The broadening use of nanomaterials calls for the regulatory body to be up to the task and to develop policies that strike a balance between the benefits and risks of these materials. A major direction in future policy development will be the implementation of nanomaterials within the bounds of sustainable water management. The promises of improved systems should not come at the expense of environmental safety. Policies should highlight sustainable production, application, and removal of nanomaterials during water treatments. Another priority for future policy development should be to consider major international guidelines on nanomaterial safety in a coordinated manner. Because nanomaterials are developed and traded across borders, international cooperation is necessary to develop regulations that protect human health and the environment [131,132].

## 7. Sustainable Strategies for Safe Nanomaterial Application

**Figure 3:**
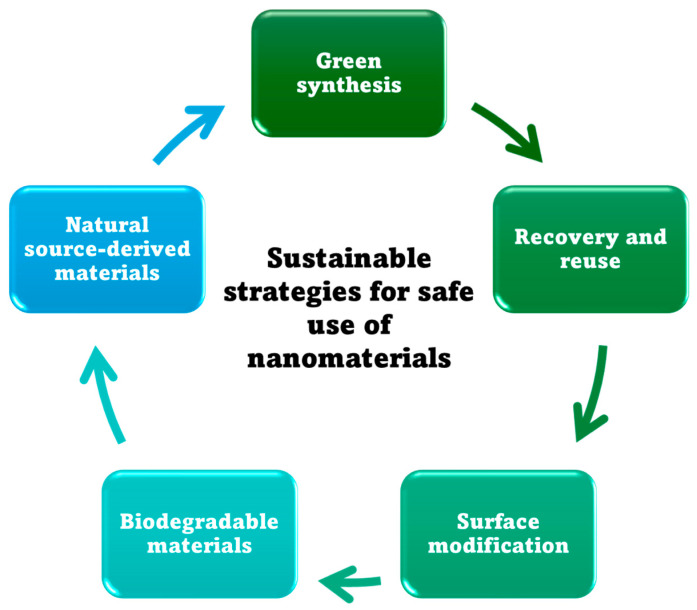
Schematic overview of sustainable strategies for safe nanomaterial use in water decontamination applications.

Nanomaterial applications have offered unlimited innovation potential in fields such as water treatment, biomedical technology, and energy. At the same time, nanomaterials pose both environmental and health risks and benefits. That is, properties enabling nanomaterials to remove pollutants, deliver drugs, and be used effectively—such as nanoscale size, high reactivity, and surface area—are also properties that make them potentially hazardous if not properly managed. Therefore, increasing attention is being paid to sustainable systems that will ensure the safe application of nanomaterials with a minimal ecological and toxicological impact [59,99,100,112,133] (Figure 3).

### 7.1. Green Synthesis and Eco-Friendly Nanomaterials

A strategic approach to ensure the safe application of nanomaterials could incorporate eco-friendly nanomaterials and green synthesis. The term “green synthesis” refers to the method of producing nanomaterials through non-toxic processing techniques, using raw materials that do not contain hazardous elements and, on a larger scale, require minimal high-energy inputs. It presents an avenue for developing naturally derived materials, such as plant extracts, microorganisms, or biological molecules, for the synthesis of nanomaterials [134]. Similarly, biodegradable nanomaterials focus on developing materials that degrade naturally in the environment, leaving no harmful residues. This approach is particularly important in cases such as water treatment, which may lead to leakage of nanomaterials into natural ecosystems. Biodegradable nanomaterials, or those derived from natural sources, can help reduce prolonged buildup in soil and aquatic systems, thereby reducing the risk of bioaccumulation and negative impacts on both terrestrial and aquatic life [55,135].

### 7.2. Strategies for Reducing Environmental Toxicity

Although the intrinsic characteristics of nanomaterials contribute to their remarkable efficiency across diverse applications, they may simultaneously increase toxicity. This challenge could be addressed by modifying the surface of nanomaterials to reduce environmental toxicity. Surface modifications increase stability and decrease reactivity, thereby preventing deleterious interactions with biological systems [16,24]. Another approach would be to functionalize nanomaterials to enhance selectivity towards specific pollutants or even biological molecules. Improving targeting capabilities for nanomaterials would greatly reduce the likelihood of unintended interactions with nontarget organisms or environmental components. One emerging aspect under the umbrella of safe-by-design nanomaterials is the synthesis of inherently less toxic nanomaterials. By optimizing the shape, size, or surface charge of nanomaterials, researchers may be able to reduce adverse environmental and health effects without undermining nanomaterials’ effectiveness in their intended applications [11,25,110].

### 7.3. Nanomaterial Recovery and Reusability

An important consideration for the sustainability of nanomaterial applications is the use of strategies to prevent nanomaterials from accumulating in the environment after use. Magnetic nanoparticles should find use since they can be placed in water or soil by an applied magnetic field from the outside, hence re-collecting them for reuse in further cycles [21,35]. Therefore, carbon-based materials, such as graphene or CNTs, can be nanomodified to enable regenerative recovery via filtration or centrifugation. After regeneration, these nanomaterials can once more find their application in multiple cycles of treatment, supporting economic and environmental sustainability through waste and resource reduction [96,100]. The reusability and recycling of nanomaterials are crucial for applications such as water decontamination, since they could otherwise lead to massive pollution during large-scale implementation. By designing nanomaterials that maintain their structural integrity and effectiveness across multiple cycles, researchers will address the need for continuous production in a way that, in the end, decreases the environmental footprint of the entire nanotechnology enterprise [19,20,118].

### 7.4. Life Cycle Assessment of Nanomaterial-Based Technologies

For a balanced assessment of risks and benefits, the weight of environmental benefits by nanomaterial-based technologies requires a comprehensive life cycle assessment (LCA). Ending in their disposal after use gives a holistic perception of the environmental impacts of nanomaterials from production. It therefore evaluates all stages of the nanomaterial’s life cycle, including extraction of raw materials, synthesis, application, recovery, and disposal [132,133,134]. The LCAs identify “tipping points” where maximum environmental impacts occur; thus, in this direction, they help researchers and policymakers develop mitigation strategies. LCAs can also be used to compare nanomaterial-based technologies with traditional ones and determine whether the former provide environmental benefit over the latter. Incorporating LCA data into regulatory frameworks can also support the development of more effective policies that prioritize environmentally friendly nanomaterials and guide industries toward more sustainable practices [126,132].

## 8. Future Research Directions and Conclusions

Based on the comparative analysis presented throughout this review, among the diverse classes of nanomaterials investigated for water decontamination, polymeric nanostructures emerge as up-and-coming candidates, with the potential to reconcile high treatment efficiency with long-term environmental safety. Benefiting from intrinsic design flexibility, polymeric nanomaterials can be tailored in terms of surface chemistry, degradation behavior, and interactions with contaminants and biological systems. Compared with many inorganic and carbon-based nanomaterials, polymer-based systems offer greater opportunities to incorporate biodegradability, reduced ecotoxicity, and life-cycle considerations directly into material design.

Consequently, polymeric and polymer-based hybrid nanomaterials represent a strategic platform for the development of next-generation water treatment technologies that address both immediate remediation needs and long-term ecological sustainability.

Despite significant advances in nanomaterial design, one of the most critical gaps in contemporary research remains the need to thoroughly identify and resolve the toxicities of recently discovered nanomaterials, such as CNTs, metal oxide nanoparticles, and polymeric nanoparticles, which have remarkable efficiencies in pollutant removal but pose long-term consequences [16,24]. Explicitly, research must be designed to understand how nanomaterials interact with living organisms across diverse trophic levels, the possibilities and pathways of their bioaccumulation in ecosystems, and the potential for biomagnification, including both forms of toxicity over prolonged periods and across different environmental settings. Improvements in the performance of nanomaterials in real water-treatment applications are still needed. A number of materials have so far shown high efficiency in laboratory conditions but have not yet found their way into real-world, large-scale applications. The main focus of future research should be the study of the increased stability, reusability, and selectivity of nanomaterials to operate under all environmental conditions with no risk to ecosystems [35,37,39].

Another top research gap that could provide direction in the coming years concerns narrowing the divide between laboratory research and real-world applications. Most studies on nanomaterial-based systems for treating water rely on results from small-scale or laboratory-controlled experiments that do not reflect the complexity of natural systems. To help bridge this divide, there should be an emphasis on encouraging industry–academia partnerships to scale up implementation through pilot projects across various settings [136,137]. In addition, studies need to prioritize the development of standardized testing methodologies to assess the environmental risks posed by nanomaterials. At the moment, there is a general lack of harmonized toxicology testing protocols, which makes it difficult to compare results and assess the actual risk levels of nanomaterials to water de-pollution. Globally accepted standards are critical to both regulatory approval and wider adoption of nanomaterials in environmental applications [138].

Efficiency and environmental and human health protection should be balanced in future research and development, with a focus on the effective and sustainable use of nanomaterials. This balance can be achieved by emphasizing high pollutant-removal performance while ensuring that nanomaterial deployment does not introduce new environmental hazards [15,92]. This will indeed be possible if green synthesis methods and eco-friendly nanomaterials are used to reduce the ecological footprint of nanotechnology. In parallel, recovery technologies and reuse mechanisms for these nanomaterials must continue to be developed for water treatment systems, to minimize environmental release and secondary contamination [132,135].

Integrating life cycle assessment (LCA) data into the regulatory framework can also be seen as a mechanism for better implementing policies that prioritize the environmentally benign character of nanomaterials and directing the industry towards more sustainable practices. By considering the full life cycle of nanomaterials, from production through application to disposal, nanotechnology can deliver substantial benefits in water decontamination without compromising long-term environmental safety. Such holistic LCA-driven strategies enable the identification of environmental impact hotspots and support the development of targeted mitigation strategies, thereby reinforcing the sustainable implementation of nanomaterials in water treatment applications [126,132].

Future research should focus on actionable priorities like (i) assessing nanomaterial interactions with organisms at multiple trophic levels and mapping bioaccumulation and biomagnification pathways under realistic environmental conditions; (ii) assessing nanomaterial stability, selectivity, and reusability in complex aqueous matrices with coexisting ions and natural organic matter; and (iii) developing standardized, harmonized testing protocols. Answering these problems and technical barriers will bridge laboratory research and scalable, safe, and sustainable water treatment applications. To conclude, continued interdisciplinary efforts are essential for integrating materials science, environmental toxicology, and regulatory policy.

## Figures and Tables

**Table 5:** Summary of the fate and behavior of nanomaterials in the environment.

Aspect	Description	Influencing Factors	Environmental Impact	Refs.
Transport and Mobility	Once released into aquatic systems, nanomaterials undergo physical and chemical processes like dispersion, aggregation, sedimentation, and dissolution that govern their mobility and behavior.	Nanoparticle physicochemical properties (size, shape, surface charge), water flow dynamics (turbulence), temperature, and the presence of solutes or other particles.	Determines how far nanoparticles travel, their potential for exposure to aquatic life, and their interaction with pollutants.	[2,31]
Dispersion	Refers to the distribution of nanoparticles throughout a water body. Uniform dispersion increases potential contact with various environmental components.	Smaller particles with low density and high surface charge resist settling and are more likely to remain suspended; turbulence enhances mixing and distribution.	Increased dispersion extends the spatial reach of nanoparticles, potentially leading to greater environmental exposure and interactions with biota.	[31,103]
Aggregation	The process by which individual nanoparticles cluster into larger units due to attractive forces, often resulting in reduced stability and increased size.	Surface charge (zeta potential), ionic strength of water, pH, presence of NOM, and nanoparticle hydrophobicity.	Aggregated nanoparticles are less mobile, more prone to sedimentation, and may exhibit reduced reactivity with contaminants.	[104,105]
Sedimentation	The settling of nanoparticles or their aggregates to the bottom sediments of water bodies due to gravity.	Particle size and density, degree of aggregation, and water column turbulence or flow rate.	Leads to the accumulation of nanoparticles in benthic zones where they may persist, become buried, or be resuspended under disturbed conditions.	[106,107]
Dissolution	Nanoparticles disintegrate or dissolve into their ionic forms, often increasing their bioavailability and changing their environmental behavior.	Affected by pH (acidic conditions accelerate dissolution), ionic strength, and the presence of complexing agents like NOM.	Can lead to increased toxicity by releasing bioavailable ions, altering the nanoparticles’ risk profile.	[37,38,43,54,83]
NOM Interaction	Nanoparticles interact with NOM through adsorption, surface coating, or complexation, altering surface chemistry and stability.	Composition and concentration of NOM, nanoparticle surface properties, and environmental conditions (pH, ionic strength).	Can either stabilize nanoparticles by steric repulsion or induce aggregation; also affects pollutant removal efficiency due to site blocking or co-adsorption.	[24,28,38,82,98,108,109,110]
Oxidation	Nanoparticles undergo chemical transformation in oxidizing environments, forming oxides or releasing metal ions.	Presence of oxygen, the redox potential of the environment, and nanoparticle composition	May increase toxicity but can also passivate surfaces and reduce reactivity over time.	[35,36,111]
Sulfidation	Reaction of metal-based nanoparticles with sulfur-containing compounds leads to the formation of metal sulfides.	Availability of sulfides in sediments or wastewater and metal reactivity.	Produces less soluble, less toxic forms of metals, decreasing environmental risk but potentially reducing remediation performance.	[36,57,112]
Photoinduced Transformation	Light exposure, especially UV, can drive chemical changes in nanoparticles, such as surface oxidation or ROS generation.	Solar radiation intensity, wavelength, and nanoparticle type; presence of sensitizers or photocatalysts.	Can increase or decrease toxicity/reactivity, influence pollutant degradation, and alter nanoparticle surface chemistry over time.	[95,113]

Abbreviations: NOM—natural organic matter.

## Data Availability

Not applicable.
